# Validation of various adaptive threshold methods of segmentation applied to follicular lymphoma digital images stained with 3,3’-Diaminobenzidine&Haematoxylin

**DOI:** 10.1186/1746-1596-8-48

**Published:** 2013-03-25

**Authors:** Anna Korzynska, Lukasz Roszkowiak, Carlos Lopez, Ramon Bosch, Lukasz Witkowski, Marylene Lejeune

**Affiliations:** 1, Nalecz Institute of Biocybernetics and Biomedical Engineering, Ks. Trojdena 4 Str., Warsaw, Poland; 2, Molecular Biology and Research Section Hospital de Tortosa Verge de la Cinta, C Esplanetes 14, Tortosa, Spain

**Keywords:** Lymphoma, Immunohistochemistry, Nuclear quantification, Pathology, Morphometry

## Abstract

The comparative study of the results of various segmentation methods for the digital images of the follicular lymphoma cancer tissue section is described in this paper. The sensitivity and specificity and some other parameters of the following adaptive threshold methods of segmentation: the Niblack method, the Sauvola method, the White method, the Bernsen method, the Yasuda method and the Palumbo method, are calculated. Methods are applied to three types of images constructed by extraction of the brown colour information from the artificial images synthesized based on counterpart experimentally captured images. This paper presents usefulness of the microscopic image synthesis method in evaluation as well as comparison of the image processing results. The results of thoughtful analysis of broad range of adaptive threshold methods applied to: (1) the blue channel of RGB, (2) the brown colour extracted by deconvolution and (3) the ’brown component’ extracted from RGB allows to select some pairs: method and type of image for which this method is most efficient considering various criteria e.g. accuracy and precision in area detection or accuracy in number of objects detection and so on. The comparison shows that the White, the Bernsen and the Sauvola methods results are better than the results of the rest of the methods for all types of monochromatic images. All three methods segments the immunopositive nuclei with the mean accuracy of 0.9952, 0.9942 and 0.9944 respectively, when treated totally. However the best results are achieved for monochromatic image in which intensity shows brown colour map constructed by colour deconvolution algorithm. The specificity in the cases of the Bernsen and the White methods is 1 and sensitivities are: 0.74 for White and 0.91 for Bernsen methods while the Sauvola method achieves sensitivity value of 0.74 and the specificity value of 0.99. According to Bland-Altman plot the Sauvola method selected objects are segmented without undercutting the area for true positive objects but with extra false positive objects. The Sauvola and the Bernsen methods gives complementary results what will be exploited when the new method of virtual tissue slides segmentation be develop.

**Virtual Slides:**

The virtual slides for this article can be found here: slide 1: http://diagnosticpathology.slidepath.com/dih/webViewer.php?snapshotId=13617947952577 and slide 2: http://diagnosticpathology.slidepath.com/dih/webViewer.php?snapshotId=13617948230017.

## Introduction

Immunohistochemically (IHC) stained tissue samples are used by pathologists to establish the diagnosis and the prognosis and the treatment in various types of cancer [1-4]. The evaluation process takes into account the amount of immunopositive cells (membrane, cytoplasm or nuclear staining) and the architecture of the tissue sample. Such evaluation can be done by the experienced pathologist directly via microscope or from digital images of the samples.

The human direct evaluation is irreproducible, time-consuming as well as intra- and interobserver error prone [5]. So different automated methods, based on the digital image processing are proposed, as they promise the improvement of evaluation reproducibility and they can become tools for inter- and intralaboratory unification in cut-offs and threshold levels [6].

To make the validation more accurate and precise, the image segmentation should indicate cells’ membrane, cells’ cytoplasm and/or nuclei and/or other organelles (e.g. the lysosome) efficiently and robustly [7-9]. The error in objects detection ought to be as small as possible and should be given explicitly since it determines errors in features important in the process of diagnosis. Errors in objects detection influences objects morphology evaluation, pattern of objects’ distribution and texture features which reflects chromatin distribution [10,11].

Segmentation of the images of stained tissue samples is a complex problem, because of huge variability of shapes, size and colour in the objects of interest and in the general architecture of the tissue samples. So far, there have been developed many methods, which detect objects of interest in these types of images, by many groups [12-18]. These methods come from various segmentation approaches and present various advantages and disadvantages. The main obstacle is that all these methods are validated by their authors on their experimentally captured images. There is lack of any comparative study which answers a question of usefulness, efficiency and reproducibility of the particular method, applying it to the particular type of tissue and/or staining processes. Using comparative study on fixed images’ database it is possible to achieve result even if a very small difference in results of segmentation is expected.

The comparative study of results of various methods of segmentation has been performed for the fluorescent microscopy images of living cell images [6], for the stained tissue section in neuroblastoma cancer (Ki67) [8] and breast cancer cells (estrogen/progesterone status) [1]. In the case of fluorescent microscopy images segmentation, the Lehmusola and co-workers [19,20] proposed evaluate segmentation method using set of synthetic images constructed by prepared software with assumed objects’ border position. The averaged multiple manual segmentation results were treated as reference “true” in the case of the other comparisons. Because comparison results for fluorescent images allows their authors to detect small differences in method performance, it was decided to use synthetic images to compare chosen segmentation methods. This paper presents the method of artificial tissue sections images construction. In this method the position, shape and colour of objects and background are generated according to statistical model constructed based on observation the set of experimentally acquired images and on the physics of digital image acquisition and microscope image erasing. In this paper the follicular lymphoma cancer tissue sections immunohistochemically stained with 3,3’-diaminobenzidine (DAB) and contra-stained with hematoxylin (H) are under interest. The images captured from several tissue sections and from various camera and microscope sets are used to gain the knowledge about images features and characteristics.

The reliable evaluation of the chosen adaptive threshold methods of segmentation is the main goal of this investigation. The results of this study will serve as the background for developing of a new hybrid method in the next step of our investigation. But there is the additional aim of this paper: to present usefulness of the images synthesis method in evaluation and comparison of the image processing results. The synthetic images maintain features of experimental images such as level of noise, range of colour and tones, vignietting, and so on in controlled degree what gives researcher possibility to observe the influence of all the features and each feature separately on the result of image processing methods.

Next section “Related works” shows the review of principia of the automated approaches developed so far and used for various types of cancer tissue sections evaluation. The following section contains description of the characteristics of experimentally acquired lymphoma section tissue images stained with DAB & H. In total, six methods of segmentation are introduced in the other section. The experimentally collected and synthesized artificial images are presented subsequently. The validation of the methods and the results of their comparison are described in the section entitled “The results of the adaptive threshold method comparison”. The discussion and conclusions are presented in the last section.

### Related works

First systems for microscopic image analysis in histopathology, e.g. iPATH or UICC-TPCC [21], have been established as academic projects. The following steps have been performed in these systems: sampling, segmentation and calculation of chosen features which are determined among normal, benign and malignant cells or cells’ nuclei. The System EAMUS [22] followed the systems described above. It was dedicated to the digitalized glass slides, called virtual slides, for telemedicine which was designed as remote systems connected by internet with automatic image measurement systems to consults physicians and scientists. Its successor was developed under MATLAB and Java platform by Markiewicz [23] as a system for specific markers and pathologies. Both these systems are applied within the telepathology projects framework as a tool for verification the idea of the examination of microscopic images from a distance. Next semi-automatic computer-assisted systems for histopathology and immunohistopathology have become commercially available from DAKO and Aperio. But they are used as the virtual multiresolution slides constructors rather, than the sample or object in sample classification systems. More oriented towards feature evaluation is system proposed by Bueno [24] as parallel solution for high resolution histological and immunohistchmemicaly stained tissue section images. What was learned from the use of all systems described above is that the automatic image segmentation, as the bottle neck of the computer-aided image analysis method is the most complex and challenging step in both histopathological samples images of paraffin tissue sections and also for cytological smears [25-27]. There are some complex and sophisticated algorithms [8,12,14,28-30], which have been developed and tested for various markers used in digital images of the histopatological samples apply to various tissues in various pathology. All of them use various threshold methods on selected or modified colour information separated from RGB digital images. Some of them use blue channel only as it gives greatest contrast between brown and blue but loose information about brown colour spread in G an R channels [31], the other propose combination of all channels of RGB as: -“brown axis” = B-0.3*(R+G) Tadrous 2010, [32], -colour deconvolution in which three well defined colour vectors, describing new colours in old colour space, should be achieved as calibration information (Ruifrok and Johanston 2001) [33-35], -“de-staining” algorithm separating up to three visually distinct colours to effect selective contrast [32]. Minority of algorithms uses HSV colour model in which detection of the brown colour can be simply rotation of the hue axes by Kuse [36]. All of threshold methods suffer from a lack of universality as they are adjusted by specifics image parameters: level of contrast [37-39] or degree of saturation [8] and so on. It is observed that changes in image characteristics caused by tissue variability or more often by optics and camera settings cases moderate results of segmentation [15,40]. This paper compares the results of chosen thresholding methods applied to three types of colour information captured form RGB digital images: (1) B channel, (2) brown axis and (3) deconvolution to separate brown channel. It allows us to analyze which thresholding method is effective towards which type of colour information if brown objects in DAB & H staining lymphoma tissue section should be selected.

### The characteristics of experimentally acquired lymphoma section tissue images stained with DAB & H

**Figure 1:**
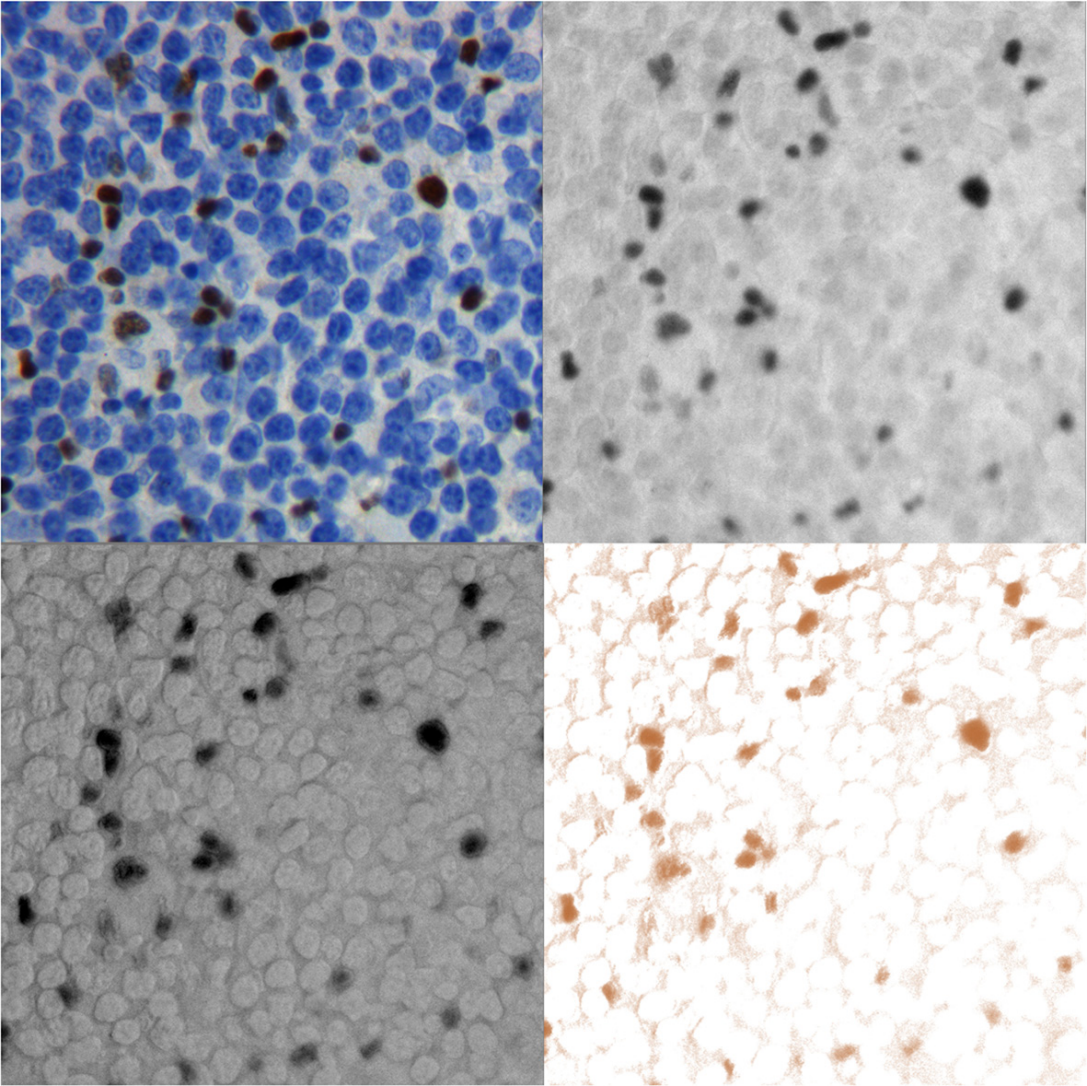
**Experimentally collected image.** The experimentally collected image (top-left) and its B-channel of RGB (top-right), its “brown” axis (bottom-left) and its brown map after colour deconvolution (bottom-right).

Digital images of tissue section of paraffin embedded lymphomas where captured in a brightfield microscope. These images differ in colour ranges, pattern of object - cells’ nuclei - distribution as well as in local and global contrast and brightness. Figure 1 (top-left) shows the image collected in the Hospital de Tortosa Verge de la Cinta using the indirect immunohistochemical primary antibodies against FOXP3 and the secondary antibodies which include the peroxidase block, labeled polymer, buffered with substrate/DAB+ chromogen and finally contra-stained with hematoxylin. All images show the brown end products for the immunopositive cells’ nuclei among blue colour nuclei for the immunonegative cells.

The singular brown objects as well as the small clusters of brown objects, surrounded by blue ones, are observed in images. Nuclei are touching, not overlapping one another, in the clusters. Variation in blue and brown colours, as well as variation in objects density in one image and from one image to another, is observed. The inside of brown objects is visible as almost homogeneous, with smooth and slightly visible texture, while the inside of blue objects seems to be filled mostly with curly texture. Cells’ nuclei marked with FOXP3 are nuclei of regulatory T-cells, it means immune system cells, so their distribution of size is similar to normal T-cells’ population (distribution with small range and sharp peak), while distribution of most of the blue nuclei cells’ population is typical for cancer cells’ population (tumoral B lymphocytes). But some image features hinder the segmentation process, e.g. a presence of: 

•spurious stain deposits in other types of cells: stromal, scar, lymphocytes;

•very dark parts of blue stained nuclei;

•partly blurred nuclei border with the colour rim caused by the chromatic aberration;

•colour noise.

Some non-homogeneity of light distribution in a single image is observed: the middle part is brighter than the peripheral one. Even images collected by one pathologist, using the particular microscope and camera, differ one from another. It is caused by random changes in external light conditions and chosen parameters of image acquisition.

All features of images and objects of interest described above, observable in Figure 1 (top-left), cause that adaptive threshold methods of segmentation are adequate to the situation. Six adaptive methods of threshold, locally adjusted to the contrast, originally defined for documents and the text segmentation, have been adjusted to analyze three versions of colour information extracted from images with objects in various shades of brown among blue textured spots on the off-white background. The chosen threshold methods, the method of comparison and the results of thresholds are presented in the next sections.

## Methods

### The chosen methods of segmentation

**Figure 2:**
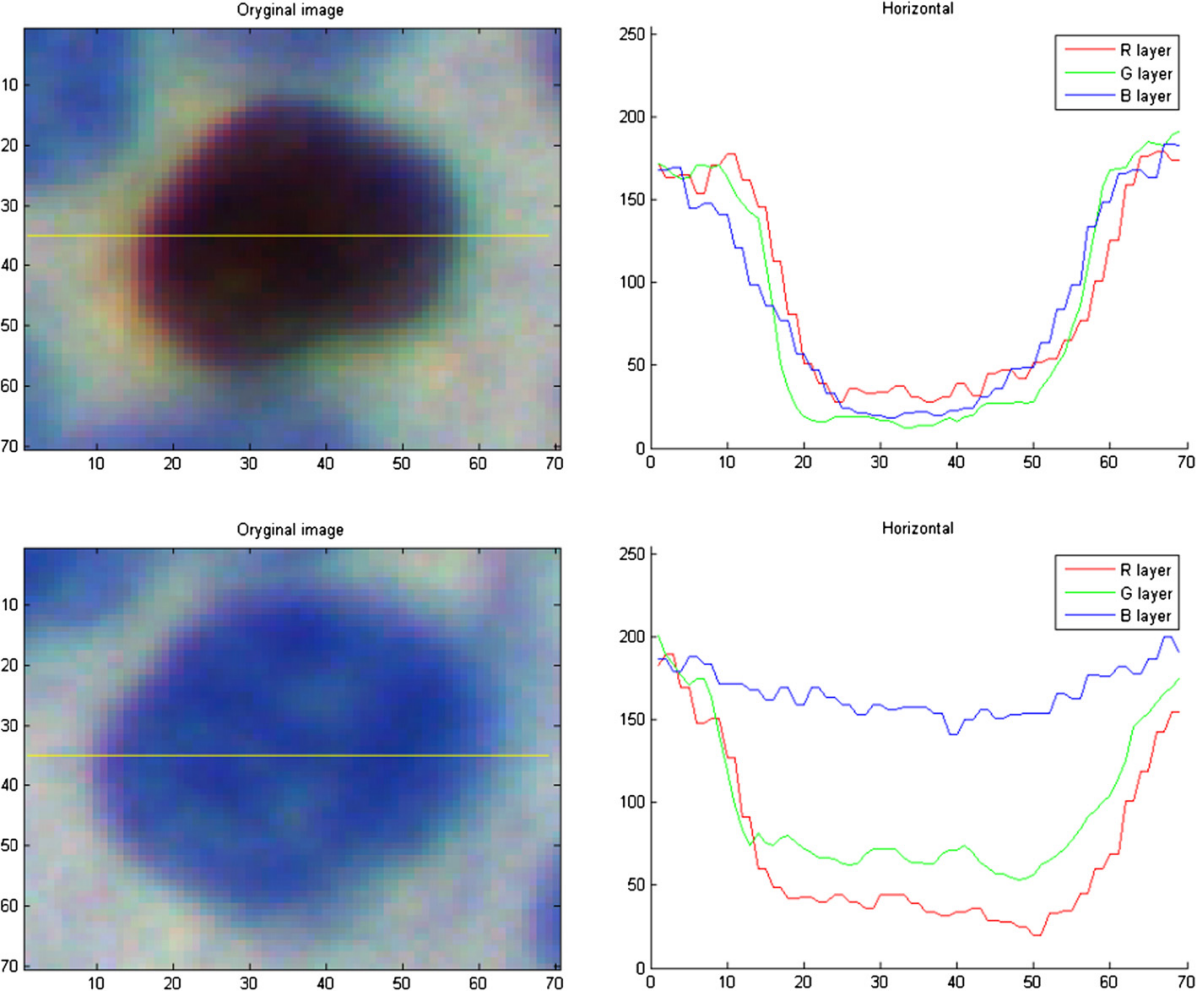
**Comparison of blue and brown objects.** The presentation of magnified brown object from experimentally collected image (top-left) and its line profile (top-right) and the blue object (bottom-left) with its line profile (bottom-right).

Image segmentation can be considered as the process of dividing an image into multiple components [41,42]. It is usually used to separate objects from the background. There are many forms of image segmentation: thresholding, clustering, transform and edge or texture based methods. The segmentation as some delimitation of boundaries between compartments in this case is limited to detect a hypothetical (not existing in real word) line between nucleus and surrounding cytoplasm or stroma. Because of contrast fluctuation between objects of interest and background across image plane and from image to image the locally adaptive thresholding methods seems to be appropriate. The method which have been defined for text detection in scanned digital documents deal with grayscale images with Gaussian and uniform noise characteristics and with big contrast. Although the acquired images are 3-channel RGB images, the segmentation algorithms treat separately monochromatic images containing separated information of brown colour: 

•the blue channel from RGB, presented in Figure 1 (top-right), because of the results of the analysis of cells’ nuclei profiles presented in Figure 2;

•the “brown channel” calculated from RGB image which is presented in Figure 1 (bottom-left);

•the results of brown colour deconvolusion from RGB image which can be observed in Figure 1 (bottom-right).

All images which have been prepared to the comparison are transformed to obtain introduced three versions of each image. All tested methods are implemented in MATLAB [23] and used to calculate results of segmentation for all version of colour information.

### Locally adaptive thresholding

Local threshold is calculated at every point of image with sliding window image processing. Threshold value is based on the intensity of the pixel and its neighborhood [43]. In this paper it is considered: two local variance methods, three local contrast methods and one center-surround scheme. All expressions used in algorithms presented below are described in Table 1.

**Table 1:** Expressions

**Expession**	**Definition**
*I*	Image of size *M*×*N*
*I*(*x*,*y*)	Intensity value
*B*(*x*,*y*)	Binarization algorithm
*T*(*x*,*y*)	Threshold value algorithm
*m*_*nb*_(*x*,*y*)	Mean value of the neighborhood of analyzed pixel
*m*_*wxw*_(*x*,*y*)	Mean value of the window of size *w*
*σ*(*x*,*y*)	Standard deviation
*μ*(*x*,*y*)	Variance
*C*(*x*,*y*)	Contrast as a difference between max and min
0	Object
1	Background

Used expressions.

#### Niblack

The most basic adaptive threshold method is Niblack method [44] and it belongs to the group of local variance methods. Local threshold is calculated based on mean and standard deviation of local neighborhood of size set by the parameter *w*. Another applied parameter *k* introduces bias of variance value.

(1) $$\begin{array}{cc} B(x,y) & =\left\{\begin{array}{ll} 1 & \text{if}\;I(x,y)>T(x,y),\\ 0 & \text{otherwise}. \end{array}\right)\text{, where}\\ T(x,y) & =m_{w\times w}(x,y)+k\cdot \sigma_{w\times w}(x,y) \end{array}$$

These two parameters of Niblack method values and the rest of used parameters values are presented in Table 2.

**Table 2:** Parameters

	***w***	***k***	***R***	***bias***	***T***_***c***_	***T***_**1**_	***T***_**2**_	***T***_**3**_	***T***_**4**_
Niblack	51	-0.2			150				
Sauvola	51	-0.2	128		150				
White	51			2	150				
Bernsen	51				150				
Palumbo	51				150	20	0.85		
Yasauda	51				150	50	100	128	15

Values of parameters used in segmentation methods.

#### Sauvola

Method presented by Sauvola and Pietaksinen [45] is another local variance method and can be treated as modified version of Niblack’s local variance method. It is based on one more parameter (*R*) which introduces the variance standardization value.

(2) $$\begin{array}{ll} B(x,y) & =\left\{\begin{array}{ll} 1 & \text{if}\;I(x,y)>T(x,y),\\ 0 & \text{otherwise}. \end{array}\right)\text{, where}\\ T(x,y) & =m_{w\times w}(x,y)+\left\{1+k\cdot \left[\frac{\sigma_{w\times w}(x,y)}{R}-1\right]\right\} \end{array}$$

#### White

The method presented by White and Rohrer [37] separates objects from background if the value of the analyzed pixel multiplied by the bias parameter is greater than mean value of neighborhood it is considered as an object. Basically, if the pixel is considerably darker than its surrounding, it is considered as an object.

(3) $$B(x,y)=\left\{\begin{array}{ll} 1 & \text{if}\;m_{w\times w}(x,y)<I(x,y)\cdot \text{bias,}\\ 0 & \text{otherwise}. \end{array}\right)$$

#### Bernsen

Another local contrast method is offered by Bernsen [38], as two stage method. Contrast value as a difference between the maximum and minimum value in neighborhood is calculated during first stage of calculation. In second stage threshold value is calculated as a mean of the minimum and maximum value in neighborhood of the analyzed pixel if the contrast value was high enough (over assumed *T*_*c*_ value).

(4) $$\begin{array}{ll} B(x,y) & =\left\{\begin{array}{ll} 1 & \text{if}\;I(x,y)>T(x,y),\\ 0 & \text{otherwise}. \end{array}\text{, where}\right)\\ T(x,y) & =\left\{\begin{array}{ll} \frac{\underset{w\times w}{\max}(x,y)+\underset{w\times w}{\min}(x,y)}{2} & \text{if}\;C_{w\times w}(x,y)\geq T_{c}\text{,}\\ 0 & \text{otherwise}. \end{array}\right) \end{array}$$

#### Yasuda

The Yasuda, Dubois and Huang’s method [39] is local contrast method and consists of four steps [46]. **Step 1.** Increasing dynamic range in the image.

(5) $$I_{1}(x,y)=\frac{I(x,y)-\min(I)}{\max(I)-\min(I)}$$

**Step 2.** Nonlinear smoothing. Replace pixel with average value (*m*_*nb*_) of its (3 by 3) neighbourhood if local range is below assumed value of *T*_1_.

(6) $$I_{2}(x,y)=\left\{\begin{array}{ll} m_{\text{nb}} & \text{if}\;(\max(\text{nb})-\min(\text{nb}))<T_{1},\\ I_{1}(x,y) & \text{otherwise}. \end{array}\right)$$

**Step 3.** Primary thresholding with course marking of background. For every pixel its neighborhood is taken and if its local contrast is not greater than assumed value of *T*_2_ or value of the pixel is greater than average of neighborhood. Wherever condition is met, it is flagged as background. For every other pixel the given calculation is performed.

(7) $$\;\begin{array}{ll} I_{3}(x,y) & \;=\;\left\{\begin{array}{ll} \;1 & \;\text{if}\;m_{w\times w}(x,y)\;<\;I_{2}(x,y)\;\vee \;c_{w}\;<\;T_{2}\\ \;\frac{I_{2}(x,y)-\underset{w\times w}{\min}(x,y)}{c_{w}} & \;\text{otherwise}. \end{array}\right)\\ \text{where}\;c_{w} & \;=\;\underset{w\times w}{\max}(x,y)-\underset{w\times w}{\min}(x,y) \end{array}$$

**Step 4.** Secondary thresholding with precise segmentation to classify rest of the pixels. Sliding window image processing uses 3 by 3 window. In this step the pixel is marked as background if minimum from neighborhood is not greater than assumed value of *T*_3_ or variance is greater than assumed value of *T*_4_.

(8) $$B(x,y)=\left\{\begin{array}{ll} 0 & \text{if}\;\underset{3\times 3}{\min}(x,y)<T_{3}\vee \sigma_{w\times w}(x,y)>T_{4}\\ 1 & \text{otherwise}. \end{array}\right)$$

#### Palumbo

The last but not least tested method designed by Palumbo, Swaminathan and Srihari [47] is using center-surround scheme. The sliding window is divided symmetrically into 9 smaller windows, but only 5 of those are used in computations. *A*_*center*_ is near neighborhood and 4 diagonal windows are far neighborhood (*A*_*neigh*_). The tested pixel is supposed to be treated as object when the central window contains the foreground object and the neighboring windows are filled with background.

(9) $$\;\begin{array}{c} B(x,y)=\left\{\begin{array}{ll} 0 & \text{if}\;I(x,y)<T_{1}\vee T_{2}\cdot m(A_{\text{neigh}})>m\cdot (A_{\text{center}}),\\ 1 & \text{otherwise}. \end{array}\right) \end{array}$$

### Hybrid methods

Niblack and Sauvola methods appear to be insufficiently sensitive in case of ICH images and they were modified for a better use. It was done by adding the contrast condition similar to that defined in Bernsen method.

#### Hybrid of Niblack and Bernsen

Under the contrast condition defined by Bernsen method the threshold value is calculated using the equation defined by Niblack method.

(10) $$\begin{array}{ll} B(x,y) & =\left\{\begin{array}{ll} 1 & \text{if}\;I(x,y)>T(x,y),\\ 0 & \text{otherwise}. \end{array}\right)\text{, where}\\ T(x,y) & =\left\{\begin{array}{ll} m_{w\times w}(x,y)+k\cdot \sigma_{w\times w}(x,y) & \text{if}\;C_{w\times w}\geq T_{c},\\ 0 & \text{otherwise}. \end{array}\right) \end{array}$$

#### Hybrid of Sauvola and Bernsen

Under the contrast condition defined by Bernsen method the threshold value is calculated using the equation defined by Sauvola method.

(11) $$\;\begin{array}{ll} B(x,y) & =\left\{\begin{array}{ll} 1 & \text{if}\;I(x,y)>T(x,y),\\ 0 & \text{otherwise}. \end{array}\right)\text{, where}\\ T(x,y) & =\left\{\begin{array}{ll} m_{w\times w}(x,y)\;+\;\left\{1\;+\;k\cdot \;\left[\frac{\sigma_{w\times w}(x,y)}{R}\;-\;1\right]\right\} & \;\text{if}\;C_{w\times w}\geq T_{c},\\ 0 & \;\text{otherwise}. \end{array}\right) \end{array}$$

From this point onward, reference to the Niblack and Sauvola methods means their respective Hybrids with Bernsen method. After a successful segmenting the image, a simple postprosessing is done. The used postprocessing consist of tresholding by size where every object with area lesser than 900px is discriminated from outcome image.

### The methods of comparison of the chosen segmentation methods results

Testing synthetic images were paired with their corresponding binary representation (template) where assumed shape and location of positive cells’ nuclei are marked. Taking into account the binary image as a reference following measurements are possible: - true positive (TP), - true negative (TN), - false positive (FP), - false negative (FN), basing on template and results of each segmentation method.

Based on these parameters, statistical measurement of the performance of segmentation methods can be calculated:

Sensitivity

(12) $$S=\frac{\text{TP}}{\text{TP}+\text{FN}}$$

Specificity

(13) $$P=\frac{\text{TN}}{\text{TN}+\text{FP}}$$

Dice’s coefficient

(14) $$r_{D}=\frac{2\cdot \text{TP}}{2\cdot \text{TP}+\text{FN}+\text{FP}}$$

Jaccard’s coefficient

(15) $$r_{J}=\frac{\text{TP}}{\text{TP}+\text{FN}+\text{FP}}$$

Sokal and Sneath’s coefficient

(16) $$r_{\text{SS}}=\frac{\text{TP}}{\text{TP}+2\cdot \text{FN}+2\cdot \text{FP}}$$

Rogers and Tanimoto’s coefficient

(17) $$r_{\text{RT}}=\frac{\text{TP}+\text{TN}}{\text{TP}+\text{TN}+2\cdot \text{FN}+2\cdot \text{FP}}$$

To analyze agreement between results of segmentation and ‘true’ value presented by template the Bland-Altman plots (B-A plots) were produced for 70 objects segmented for each method (6) and each type of colour information (3) and for selected feature (5) e.g. area, axis ratio of the ellipse fitted to object, roundness, solidity and eccentricity. The results of the analysis of 90 plots encouraged us to develop our own parameter which allows us to find any bias or presence of outliners in cretin aspect of method performance. This parameter was defined as the sum of false positive (FP) and false negative (FN) areas divided by area of ‘true’ object observed in the function of distance between centroids of the ’true’ objects and segmented object. Plots similar to B-A plot but comparing the centroids distance with the sum of FP and FN divided by area of ‘true’ object allow identification of objects with specifically distributed erroneously detected pixels. When the distance between centroids has small value while second parameter has big value it means that extra detected or undetected area is homogeneously distributed around the object otherwise badly detected or undetected area is located in such a way that detected area centroid moves away from template object centroid. It allows us to determine if any of examined methods presents any stable or occasionally occurring bias in erroneously detected area.

## The experimentally collected and the synthesized artificial images

### The experimentally collected images

The variability in appearance of the tissue section in images stained with DAB & H is remarkable due to: (1) inherent features of tissue and variability of morphology in pathological cases, (2) inherent variability of results of the staining process and (3) inherent microscopic deformations as well as introduced artefacts and noise.

The morphology of pathological follicular lymphoma tissues varies [48]. Besides the different pathological manifestations, the variability in appearance of staining samples increases during the tissues preparation. This procedure is standardized but has a non-deterministic nature because the number of chemical particles of the stain bound to the nucleus is random. It implicates variation in the brown colour, from the intensive orange, through the intensive brown to the dark brown in immunopositive nuclei [8,14]. The paper deals with samples immunohistochemically stained against FOXP3, which indicates nuclei of regulatory T-cells [3]. This type of staining procedure produces brown objects (immunopositive nuclei of regulatory T-cells) among blue objects (mostly immunonegative nuclei of tumoral B lymphocytes). Examination of the lymphoma samples leads to score the number of regulatory T-cells in the cancer tissue, what allows estimating this specific organism’s immune response to this type of cancer.

In case of automated evaluation of tissue samples, the image acquisition should be done. Because of chosen microscope and camera settings: white balance, brightness, contrast and inherent inhomogeneity in light distribution, as well as some obstacles in the light path and noise added by microscope and camera [31], variability in nuclei appearance increases. Experimentally collected images have been acquired via a brightfield microscope (Leica DM LB2 upright light microscope, Leica Microsystems Wetzlar GmbH, Wetzlar, Germany), with 40x plane-apochromatic objective of numerical aperture 0,63. 60 images captured by the experienced pathologist from 60 areas of various complexity of the several samples have been collected in Tortosa hospital. 5 images, randomly chosen from the experimental data, have been used as the models to construct their synthetic counterparts.

### The synthesized artificial images

To compare results of any segmentation methods, the exact position of the boundary of objects should be known. Information of the nuclei position is available for artificial images, which are constructed via the simulation of the cells’ population.

The process of artificial image construction is proposed as follows: random generator chooses the position of brown and blue objects (immunopositive and immunonegative cells’ nuclei) in image plane according to the founded probability distribution of their shape and size. These distributions are estimated using collection of experimentally acquired images. The number of both types of objects, colour tones, texture of objects and background are taken from experimentally collected counterpart image as samples and numbers characteristic for the particular image. Spots of the clean background are captured to the synthetic image background layer and enlarged to form continues layer on which objects layer are located. Synthesis of objects layers is done using the adjusted version of SIMCEP software and Camera Raw 4.1 module of Photoshop CS5.

The SIMCEP, developed by Lehmussola and co-workers [19,20], is available via internet. The software is dedicated to synthesize the full colour fluorescent microscopic images of nuclei or cells’ culture. For the needs of this paper it has been adjusted to simulate images from the transmission light microscopy. The core of SIMCEP system, the generator of nuclei according to distribution of their shape and size, the template generation, the texture constructor and microscope and camera signal degradation module have been used, while problems with the specific background characteristics have been solved in Photoshop.

**Figure 3:**
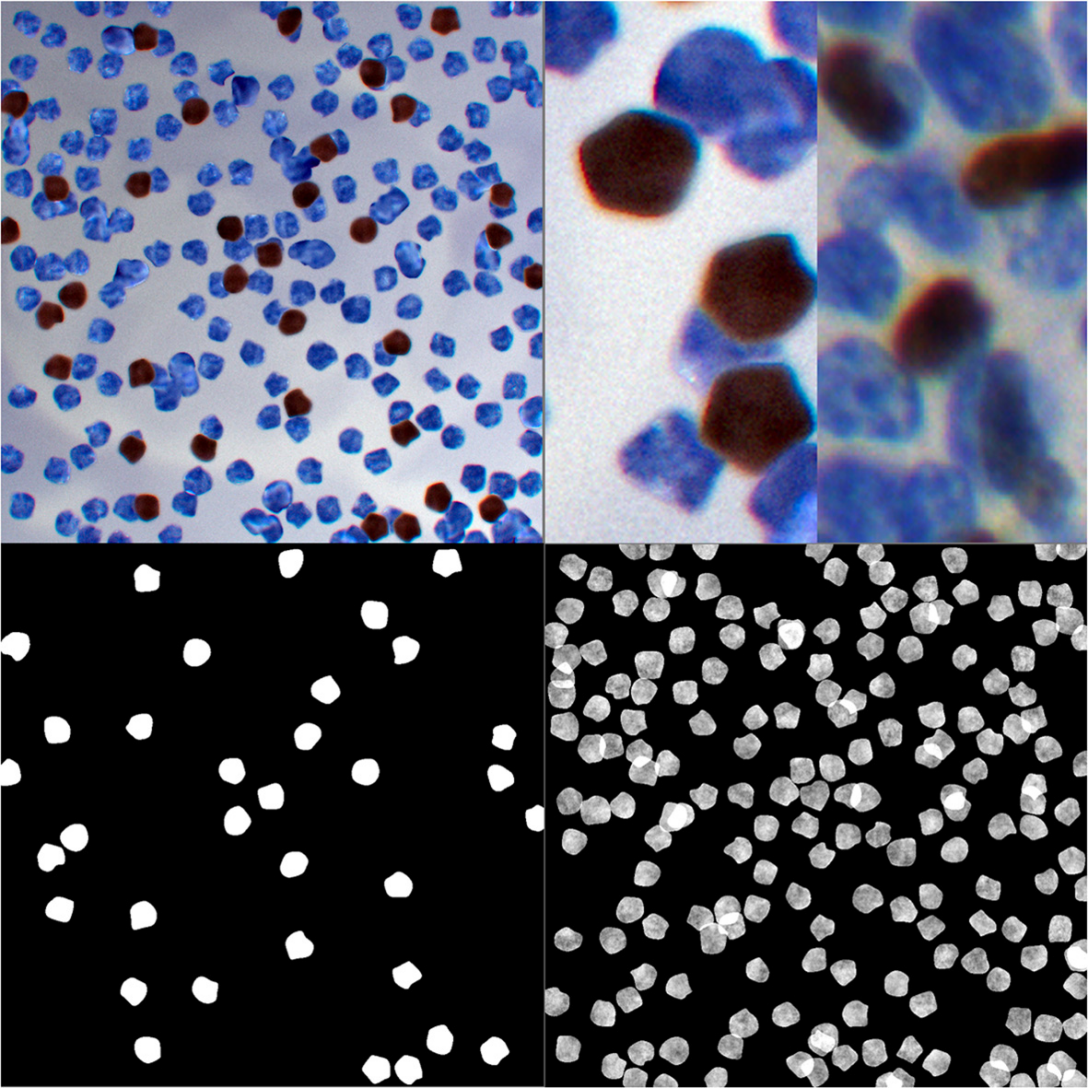
**Synthesized artificial image.** The artificial image (top-left) constructed as counterpart of image shown in Figure 1; enlarged fragment of experimentally collected image compared to the artificial image (top-right); the template of brown objects (bottom-left); the blue objects template with added Perlin texture (bottom-right).

Five experimentally acquired images of lymphoma tissue samples become the models of five artificial images, constructed as the RGB 24-bits colour synthetic microscopic images stored in uncompressed tif files. The artificial image presented in Figure 3 (top-left) has been synthesized based on the model image, presented in Figure 1 (top-left), using the template of the immunopositive cell’s nucleus position and size presented in the image in Figure 3 (bottom-left). To compare synthetic image and its counterpart image characteristic full images are presented in Figure 3 (top-left) and Figure 1 (top-left) respectively while magnified fragments of both images are presented in Figure 3 (top-right) to show details in object and background characteristics. Also, Table 3 with results of statistical comparison is provided.

**Table 3:** Objects characteristics

**Image**	**Area**		**Max radius**		**Min radius**		**Per area (obj./total)**					
	**Artificial**	**Experimental**	**Artificial**	**Experimental**	**Artificial**	**Experimental**	**Artificial**	**Experimental**				
A	1681±426	1060±413	27,4±1,4	23,3±5,0	17,4±3,9	12,9±3,2	0,0017±0,0004	0,0011±0,0004				
B	1922±166	953±406	27,9±1,3	23,5±5,9	20,7±1,8	11,7±3,4	0,0019±0,0002	0,0009±0,0004				
C	1867±256	1352±358	28,0±1,2	27,2±4,4	20,2±2,8	14,1±2,6	0,0019±0,0003	0,0014±0,0004				
D	2047±150	1078±586	29,0±1,0	24,4±6,0	21,2±1,4	11,6±4,0	0,0020±0,0002	0,0011±0,0006				
E	1892±211	892±463	27,8±1,0	21,8±6,0	20,7±2,5	9,5±4,0	0,0019±0,0002	0,0009±0,0005				

Characteristics of objects of interest (brown spots) and its distribution in experimentally collected (right column) and synthesized (left column) images.

The number of brown, marked nuclei are adjusted to the particular experimentally collected image and the templates of all nuclei location generated using SIMCEP are presented: (1) in Figure 3 (bottom-left) - immunpositive in the form of template and (2) in Figure 3 (bottom-right) - immunonegative in the form of the textured by Perlin noise map. The colours are separated form the immunopositive and immunonegative nuclei and from the background of the counterpart image after the reduction of noise and chromatic aberration in Camera Raw. All layers (background layer, brown objects of interest layer and blue nuclei layer) are put together in Photoshop. Each step of artificial image signal degradation, typical for the microscope and camera technical limitations, such as noise, vignetting and blurring, are simulated by the SIMCEP software, except of the chromatic aberration added in Camera Raw.

## The results of the adaptive threshold method comparison

**Figure 4:**
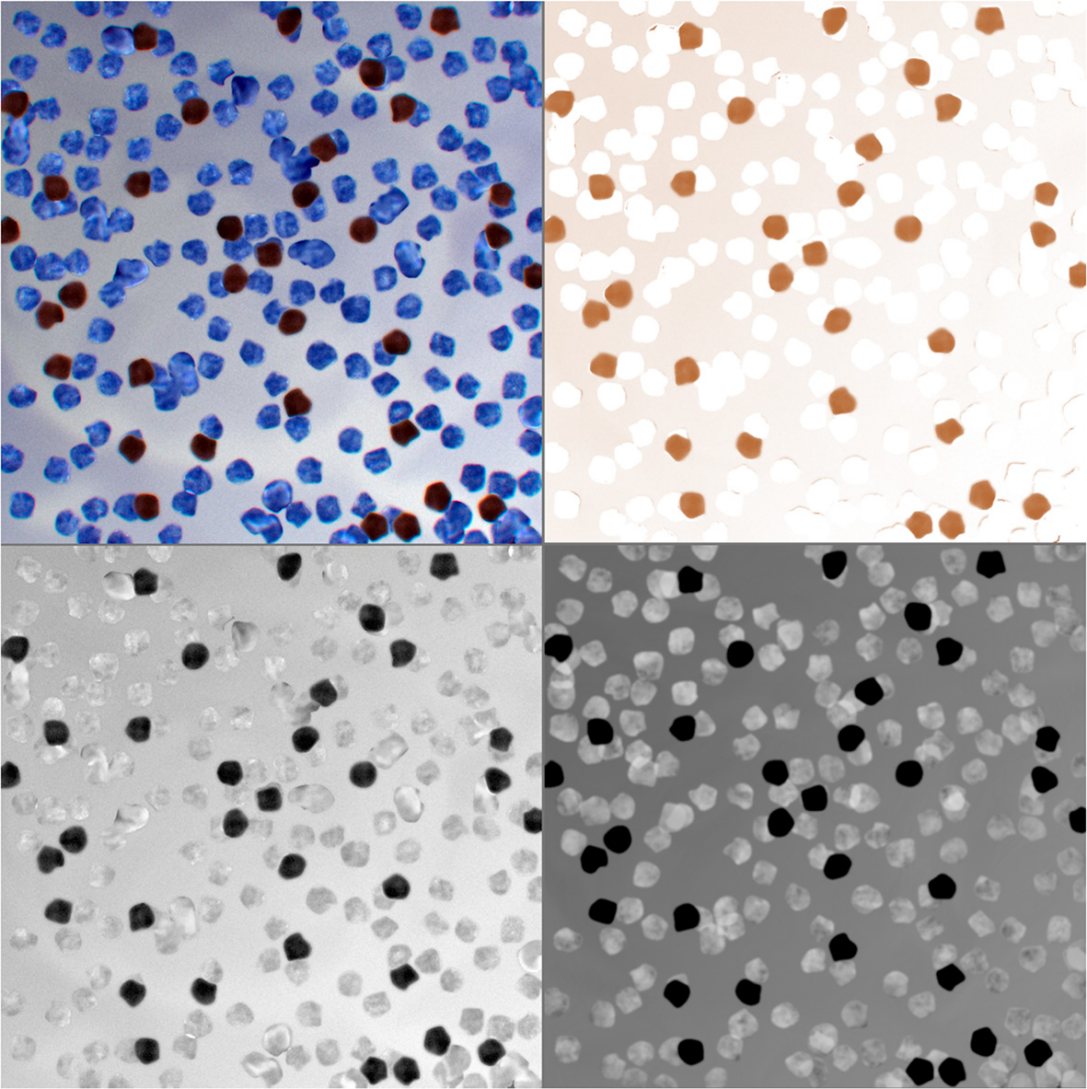
**Artificial image and three types of images calculated based on full colour image.** The artificial image (bottom-left) constructed as described in article and its B-channel of RGB (bottom-right), its “brown” axis (top-right) and its brown map after colour deconvolution (top-left).

All chosen adaptive threshold methods are applied to three types of images calculated based on full colour synthetic image (see Figure 4 top-left image): 

•B channel of RGB colour image in Figure 4 (bottom-left),

•monochromatic image calculated accordingly to the presented earlier equation as brown component extracted from all RGB channels in Figure 4 (bottom-right),

•brown part of image obtained by colour deconvolution with three colours: blue, brown and the rest called the third component in Figure 4 (top-right).

5 artificial images (from A to E) segmentation results for all objects in image (without rejection of the objects touching image border) are presented as number of found objects, the sensitivity, the specificity and four coefficients of similarity in Tables 4, 5 and 6. In Table 4 are presented results for monochromatic images constituted as B-channel, Table 5 presents results for monochromatic images constituted by deconvolution and Table 6 presents results for monochromatic images constituted as brown color extracted from RGB channels. These tables show that results for each artificial image are close for each method of segmentation applied to particular image. Generally the best results are those of segmentation applied to brown component after colour deconvolution, the mean and the standard deviation of the value of the number of found objects calculated as difference between the number of found objects and the number of ’true’ objects in template for all segmentation methods is -0.2 ±0.6 while 2.3 ±6.9 for monochromatic image with brown color extracted from RGB called ’brown channel’ and 6.0 ±13.9 for the blue channel from RGB. The mean of the sensitivity calculated for all segmentation methods is 0.9264 ±0.0611, 0.8366 ±0.1571, 0.9432 ±0.0764 respectively while mean of the specificity calculated in this data are 0.9981 ±0.0035, 0.9858 ±0.0264, 0.9886 ±0.0235. So 5 artificial images are similar one to each other and all objects in all images can be treated as homogeneous population of tested objects.

**Table 4:** The results of adaptive threshold comparison computed on channel BLUE (channel BLUE from RGB)

**Image**	**Method**	**# objects in**	**Segmented objects**	**Sensitivity**	**Specificity**	***r***_***D***_	***r***_***J***_	***r***_***SS***_	***r***_***RT***_	***M e a n *±*σ***	
		**the template**	**(filtered by size)**							**of columns *5÷10***	
A	Hyb. Niblack	8	8	0,9212	0,9998	0,9527	0,9097	0,8344	0,9975	0,9359±0,0622	
	Hyb. Sauvola	8	9	0,9443	0,9983	0,9127	0,8395	0,7234	0,9952	0,9022±0,1055	
	White	8	5	0,6461	1,0000	0,7850	0,6461	0,4772	0,9905	0,7575±0,2085	
	Bernsen	8	8	0,9447	0,9995	0,9546	0,9132	0,8403	0,9976	0,9417±0,0596	
	Palumbo	8	46	0,8632	0,9499	0,3116	0,1846	0,1017	0,9024	0,5522±0,3933	
	Yasauda	8	64	0,9981	0,8954	0,2063	0,1150	0,0610	0,8128	0,5148±0,4308	
B	Hyb. Niblack	34	34	0,9739	0,9985	0,9765	0,9540	0,9121	0,9939	0,9681±0,0317	
	Hyb. Sauvola	34	34	0,9917	0,9957	0,9658	0,9339	0,8759	0,9909	0,9590±0,0469	
	White	34	34	0,8905	1,0000	0,9421	0,8905	0,8026	0,9858	0,9186±0,0731	
	Bernsen	34	34	0,9842	0,9980	0,9782	0,9573	0,9181	0,9943	0,9717±0,0299	
	Palumbo	34	41	0,9541	0,9883	0,8992	0,8169	0,6905	0,9724	0,8869±0,1147	
	Yasauda	34	46	0,9993	0,9720	0,8328	0,7135	0,5547	0,9489	0,8369±0,1744	
C	Hyb. Niblack	15	15	0,9876	0,9997	0,9893	0,9788	0,9584	0,9988	0,9854±0,0153	
	Hyb. Sauvola	15	16	0,9929	0,9968	0,9440	0,8940	0,8083	0,9934	0,9382±0,0753	
	White	15	14	0,8392	1,0000	0,9126	0,8392	0,7230	0,9910	0,8842±0,1055	
	Bernsen	15	15	0,9873	0,9997	0,9883	0,9770	0,9550	0,9987	0,9843±0,0166	
	Palumbo	15	14	0,9280	0,9989	0,9448	0,8953	0,8105	0,9939	0,9286±0,0701	
	Yasauda	15	18	0,9998	0,9887	0,8354	0,7173	0,5592	0,9782	0,8464±0,1791	
D	Hyb. Niblack	13	13	0,9641	0,9989	0,9622	0,9272	0,8644	0,9960	0,9521±0,0504	
	Hyb. Sauvola	13	13	0,9920	0,9977	0,9549	0,9138	0,8412	0,9950	0,9491±0,0620	
	White	13	13	0,8330	1,0000	0,9088	0,8328	0,7136	0,9911	0,8799±0,1093	
	Bernsen	13	13	0,9753	0,9994	0,9761	0,9532	0,9107	0,9975	0,9687±0,0331	
	Palumbo	13	15	0,9419	0,9970	0,9187	0,8497	0,7386	0,9912	0,9062±0,0982	
	Yasauda	13	23	0,9987	0,9776	0,7090	0,5492	0,3785	0,9573	0,7617±0,2592	
E	Hyb. Niblack	21	21	0,9857	0,9995	0,9863	0,9730	0,9475	0,9978	0,9816±0,0193	
	Hyb. Sauvola	21	23	0,9937	0,9935	0,9245	0,8595	0,7537	0,9872	0,9187±0,0966	
	White	21	20	0,8386	1,0000	0,9122	0,8386	0,7220	0,9873	0,8831±0,1051	
	Bernsen	21	21	0,9871	0,9993	0,9854	0,9711	0,9439	0,9977	0,9807±0,0207	
	Palumbo	21	34	0,9422	0,9824	0,7958	0,6608	0,4934	0,9623	0,8061±0,1965	
	Yasauda	21	61	0,9975	0,9332	0,5523	0,3815	0,2357	0,8793	0,6633±0,3180	

**Table 5:** The results of adaptive threshold comparison computed on brown colour images after colour deconvolution of RGB image

**Image**	**Method**	**# objects in**	**Segmented objects**	**Sensitivity**	**Specificity**	***r***_***D***_	***r***_***J***_	***r***_***SS***_	***r***_***RT***_	***Mean±σ***	
		**the template**	**(filtered by size)**							**of columns *5÷10***	
A	Hyb. Niblack	8	8	0,9535	0,9997	0,9645	0,9315	0,8718	0,9981	0,9532±0,0478	
	Hyb. Sauvola	8	8	0,9746	0,9994	0,9646	0,9315	0,8719	0,9981	0,9567±0,0485	
	White	8	7	0,7938	1,0000	0,8851	0,7938	0,6582	0,9945	0,8542±0,1324	
	Bernsen	8	8	0,9649	0,9997	0,9702	0,9421	0,8905	0,9984	0,9609±0,0408	
	Palumbo	8	8	0,9163	0,9995	0,9373	0,8820	0,7889	0,9967	0,9201±0,0789	
	Yasauda	8	8	0,9958	0,9839	0,6270	0,4567	0,2959	0,9686	0,7213±0,3051	
B	Hyb. Niblack	34	34	0,9628	0,9999	0,9802	0,9612	0,9252	0,9949	0,9707±0,0274	
	Hyb. Sauvola	34	35	0,9805	0,9977	0,9740	0,9493	0,9034	0,9932	0,9663±0,0352	
	White	34	34	0,8828	1,0000	0,9378	0,8828	0,7902	0,9848	0,9131±0,0778	
	Bernsen	34	34	0,9648	0,9999	0,9814	0,9634	0,9294	0,9952	0,9724±0,0259	
	Palumbo	34	34	0,9445	0,9982	0,9586	0,9206	0,8528	0,9894	0,9440±0,0531	
	Yasauda	34	32	0,9984	0,9912	0,9400	0,8867	0,7965	0,9835	0,9327±0,0788	
C	Hyb. Niblack	15	15	0,9378	0,9990	0,9512	0,9070	0,8298	0,9946	0,9366±0,0629	
	Hyb. Sauvola	15	16	0,9912	0,9983	0,9671	0,9362	0,8801	0,9962	0,9615±0,0463	
	White	15	14	0,8267	1,0000	0,9051	0,8267	0,7046	0,9903	0,8756±0,1127	
	Bernsen	15	14	0,9196	1,0000	0,9580	0,9193	0,8507	0,9955	0,9405±0,0563	
	Palumbo	15	14	0,9149	0,9996	0,9489	0,9028	0,8228	0,9945	0,9306±0,0661	
	Yasauda	15	15	0,9995	0,9939	0,9046	0,8258	0,7032	0,9883	0,9025±0,1189	
D	Hyb. Niblack	13	13	0,8982	0,9994	0,9355	0,8787	0,7837	0,9934	0,9148±0,0807	
	Hyb. Sauvola	13	13	0,9228	0,9990	0,9421	0,8906	0,8028	0,9940	0,9252±0,0730	
	White	13	13	0,8047	1,0000	0,8912	0,8037	0,6718	0,9896	0,8602±0,1257	
	Bernsen	13	13	0,9019	0,9995	0,9390	0,8850	0,7936	0,9938	0,9188±0,0770	
	Palumbo	13	13	0,8788	0,9994	0,9252	0,8609	0,7557	0,9925	0,9021±0,0915	
	Yasauda	13	12	0,9469	0,9941	0,8756	0,7787	0,6376	0,9858	0,8698±0,1394	
E	Hyb. Niblack	21	21	0,9439	1,0000	0,9710	0,9436	0,8933	0,9955	0,9579±0,0398	
	Hyb. Sauvola	21	21	0,9705	0,9995	0,9789	0,9587	0,9206	0,9967	0,9708±0,0291	
	White	21	20	0,7808	1,0000	0,8769	0,7808	0,6404	0,9827	0,8436±0,1372	
	Bernsen	21	21	0,9373	1,0000	0,9676	0,9373	0,8820	0,9950	0,9532±0,0441	
	Palumbo	21	20	0,8906	0,9997	0,9381	0,8835	0,7913	0,9907	0,9156±0,0779	
	Yasauda	21	21	0,9945	0,9939	0,9288	0,8670	0,7652	0,9880	0,9229±0,0921	

**Table 6:** The results of adaptive threshold comparison computed on the “brown channel” calculated from RGB image

**Image**	**Method**	**# objects in**	**Segmented objects**	**Sensitivity**	**Specificity**	***r***_***D***_	***r***_***J***_	***r***_***SS***_	***r***_***RT***_	***Mean±σ***	
		**the template**	**(filtered by size)**							**of columns *5÷10***	
A	Hyb. Niblack	8	7	0,7310	0,9999	0,8421	0,7272	0,5714	0,9927	0,8107±0,1676	
	Hyb. Sauvola	8	7	0,7317	0,9999	0,8405	0,7248	0,5684	0,9926	0,8096±0,1686	
	White	8	8	0,9351	1,0000	0,9664	0,9349	0,8778	0,9983	0,9521±0,0463	
	Bernsen	8	7	0,7317	0,9968	0,7431	0,5912	0,4196	0,9865	0,7448±0,2242	
	Palumbo	8	12	0,9666	0,9793	0,5548	0,3839	0,2375	0,9591	0,6802±0,3313	
	Yasauda	8	20	0,9994	0,9255	0,2676	0,1545	0,0837	0,8630	0,5490±0,4230	
B	Hyb. Niblack	34	34	0,8932	0,9988	0,9353	0,8784	0,7831	0,9840	0,9121±0,0792	
	Hyb. Sauvola	34	38	0,8967	0,9926	0,8959	0,8114	0,6827	0,9731	0,8754±0,1144	
	White	34	34	0,9414	0,9997	0,9679	0,9379	0,8830	0,9919	0,9536±0,0429	
	Bernsen	34	33	0,8983	0,9813	0,8294	0,7086	0,5487	0,9529	0,8198±0,1649	
	Palumbo	34	47	0,9731	0,9569	0,7515	0,6020	0,4306	0,9193	0,7722±0,2201	
	Yasauda	34	53	0,9824	0,8919	0,5568	0,3858	0,2390	0,8146	0,6451±0,2978	
C	Hyb. Niblack	15	13	0,6038	1,0000	0,7529	0,6038	0,4324	0,9781	0,7285±0,2260	
	Hyb. Sauvola	15	13	0,6038	1,0000	0,7529	0,6038	0,4324	0,9781	0,7285±0,2260	
	White	15	14	0,8791	1,0000	0,9357	0,8791	0,7843	0,9933	0,9119±0,0817	
	Bernsen	15	13	0,6038	1,0000	0,7529	0,6038	0,4324	0,9781	0,7285±0,2260	
	Palumbo	15	17	0,9758	0,9944	0,8992	0,8168	0,6904	0,9878	0,8941±0,1208	
	Yasauda	15	24	1,0000	0,9718	0,6715	0,5055	0,3382	0,9467	0,7390±0,2775	
D	Hyb. Niblack	13	12	0,7979	0,9993	0,8757	0,7789	0,6379	0,9880	0,8463±0,1376	
	Hyb. Sauvola	13	12	0,8120	0,9985	0,8696	0,7694	0,6252	0,9871	0,8436±0,1411	
	White	13	13	0,8960	0,9997	0,9395	0,8859	0,7951	0,9939	0,9183±0,0768	
	Bernsen	13	12	0,8109	0,9989	0,8758	0,7791	0,6381	0,9878	0,8485±0,1366	
	Palumbo	13	16	0,9605	0,9948	0,8937	0,8078	0,6776	0,9879	0,8870±0,1244	
	Yasauda	13	21	0,9940	0,9744	0,6788	0,5137	0,3457	0,9511	0,7430±0,2737	
E	Hyb. Niblack	21	15	0,5377	0,9997	0,6959	0,5336	0,3639	0,9633	0,6823±0,2547	
	Hyb. Sauvola	21	15	0,5401	0,9992	0,6931	0,5303	0,3608	0,9627	0,6810±0,2553	
	White	21	21	0,8987	1,0000	0,9466	0,8986	0,8159	0,9920	0,9253±0,0691	
	Bernsen	21	15	0,5405	0,9982	0,6826	0,5182	0,3497	0,9608	0,6750±0,2588	
	Palumbo	21	26	0,9665	0,9908	0,8832	0,7909	0,6541	0,9799	0,8776±0,1332	
	Yasauda	21	44	0,9970	0,9313	0,5452	0,3748	0,2306	0,8760	0,6591±0,3202	

The next step of comparison and evaluation concerns rather methods of adaptive threshold so it have been done on the level of single object (not single image). Because objects that touch borders are segmented with holes or cavities what cause that in most cases these object disappear during the step of size filtering in further evaluation it was taking in to account only these objects which do not touching image border. As new designed method will be applied to the virtual slides which will be analysed by parallel algorithms dealing with images which are fragments of virtual slides selected with covering margins so the rejection of objects touching image border would be compensate on the level of results connection.

**Figure 5:**
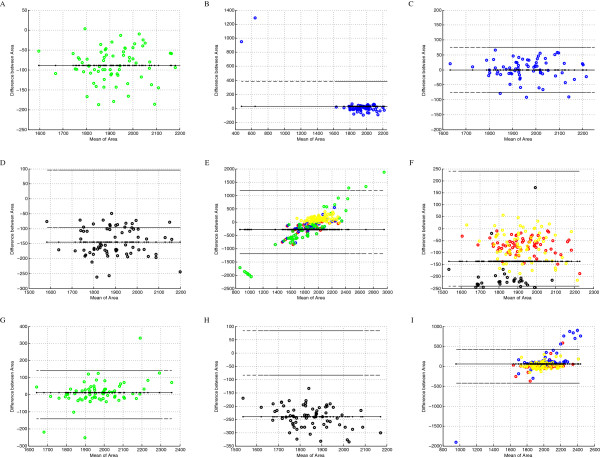
**The Bland-Altman plots of area feature.** The Bland-Altman plots of area feature. **(A)** Bernsen method of segmentation applied to image after colour deconvolution; **(B)** Sauvola method of segmentation applied to image after colour deconvolution; **(C)** Sauvola method of segmentation applied to image after colour deconvolution, presentation of true positive objects only; **(D)** White method of segmentation applied to ‘brown channel’; **(E)**Niblack, Sauvola, Bernsen and Palumbo method of segmentation applied to ‘brown channel’; **(F)** Niblack, White and Palumbo method of segmentation applied to image after colour deconvolution; **(G)**Bernsen method of segmentation applied to blue channel of RGB; **(H)** White method of segmentation applied to blue channel of RGB; **(I)**Niblack, Sauvola and Palumbo method of segmentation applied to blue channel of RGB. [colour representation: Niblack - red, Sauvola - blue, Bernsen - green, White - black and Palumbo - yellow].

**Figure 6:**
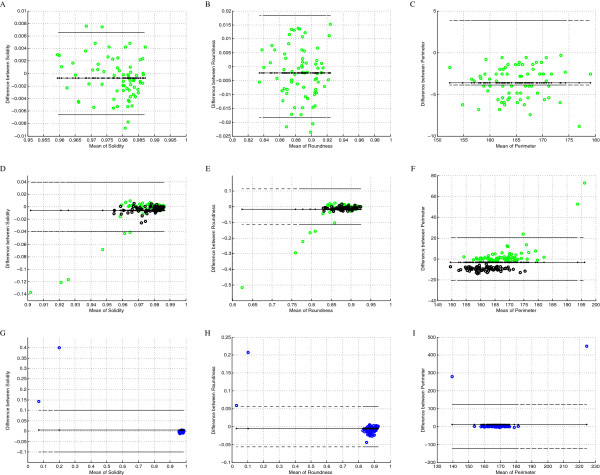
**The Bland-Altman plots of shape features.** The Bland-Altman plots of shape features. **(A)**solidity, Bernsen method of segmentation on image after colour deconvolution; **(B)**roundness, Bernsen method of segmentation on image after colour deconvolution; **(C)**perimeter, Bernsen method of segmentation on image after colour deconvolution; **(D)**solidity, White and Bernsen method of segmentation applied to blue channel of RGB; **(E)**roundness, White and Bernsen method of segmentation applied to blue channel of RGB; **(F)**perimeter, White and Bernsen method of segmentation applied to blue channel of RGB; **(G)**solidity, Sauvola method of segmentation on image after colour deconvolution; **(H)**roundness, Sauvola method of segmentation on image after colour deconvolution; **(I)**perimeter, Sauvola method of segmentation on image after colour deconvolution. [colour representation: Sauvola - blue, Bernsen - green and White - black].

The evaluation of the segmentation results of single object is presented as B-A plots for such objects’ features as area of object, roundness, eccentricity and so. The comparison of objects’ area in pixels for all except one segmentation methods (for five methods) calculated for each of 3 types of monochromatic images collecting various information about brown colour from five true colour artificial images are presented in Figure 5A-I. The Yasuda method was excluded from presentation because of its performance; it does not select certain fraction of object and at the same time it selects essential fraction of false positive objects for all types of images (for blue channel 103, for brown colour 63, for results of colour deconvolution only 2) so its plots are not presented in the paper. Some of the plots in Figure 5 (A, B, C, D, G and H) consist of about 70 non-touching image border objects from 5 synthetic images, while the others (E, F and I) present combined plots showing distinguishable by colours 3 or 4 methods’ results together. In Figure 5 and Figure 6 objects segmented by the Niblack method are presented in red, by the Sauvola method in blue, the Bernsen method in green, the White method in black and the Palumbo method in yellow.

It is visible in Figure 5 that results of almost all methods applied to images after colour deconvolution (A, B, C, F) are better than applied to blue channel of RGB (G, H, I) and to the brown component extracted from all channels of RGB (D, E); the latter seems to be the worst. Generally, it is visible that some B-A plots of area comparison between template objects and detected objects show systematic under-segmentation of area. Bernsen method (Figure 5A) and Niblack, Palumbo, and White methods (Figure 5F) applied to images after colour deconvolution and White method applied to brown component monochromatic image (Figure 5D) and to blue channel of RGB (Figure 5H) shows that there is a bias in the segmented object area. This bias is visible as objects’ area decrease in comparison to the corresponding template object area but all these method are accurate and precise in objects number. For the Bernsen method accurate and precise both are equal 1 while for the modified Sauvola method are equal 1 and 0.9722 respectively. At the same time the size of object detected by: Sauvola method applied to image after colour deconvolution (Figure 5B), Bernsen method applied to the blue channel from RGB, Palumbo method also applied to the blue channel and Yasuda method applied to all three types of monochromatic images (not presented in paper) seems not biased in objects’ area detection. But some of methods mentioned above in various degree detect extra objects in background (false positive object, FP). For the Sauvola method the number of FP objects is minimal (2 from 72) while for the Yasuda method these numbers are vast as it was mention above. These results are the reason that the Yasuda method is excluded from further consideration. To find method which is accurate enough in area detection the comparison as B-A plots, between area of the segmented and the ‘true’ object from template, is done. The difference between area of the segmented and the ‘true’ object from template for the Sauvola method applied to the result of image deconvolution for all selected object (Figure 5B) are ranged between -100 to 1400 pixels and for true positive objects only (Figure 5C) between ±80 pixels while the Bernsen method applied to blue channel of RGB (Figure 5G) and the Palumbo method (yellow circles in Figure 5F) applied to blue channel of RGB are ranged in ±130 pixels and ±170 pixels. So the error in area detection is the lowest if the objects are selected by the Sauvola method but only if false positive object are excluded based on the other information.

To reject extra objects selected by the Sauvola method two sources of information could be used: - from biased in object size segmentation method which produce accurate and precise result in number of detected objects so these results can be used to mark true positive object among the Sauvola method results or - from objects found by the Sauvola method can be filtered by any or by all of described below shape coefficients classifier.

To find segmentation method that gives precise number of detected objects and at the same time decrease objects’ size by homogeneous area rejection around objects’ periphery, only methods applied to image after colour deconvolution (Figure 5A,F) or blue channel (Figure 5G,H,I) should be taken into consideration. B-A plots for the area feature for monochromatic image from brown color extracted from RGB (Figure 5E) shows rather biased results (from -100 to -350 pixels) because of presence of cavities and holes in large fraction of segmented objects. So the following three methods: the Bernsen method applied to the results of colour deconvolution (Figure 5A) and to blue channel of RGB (Figure 5G) and the White method applied to blue channel (Figure 5H) are taken into consideration.

**Figure 7:**
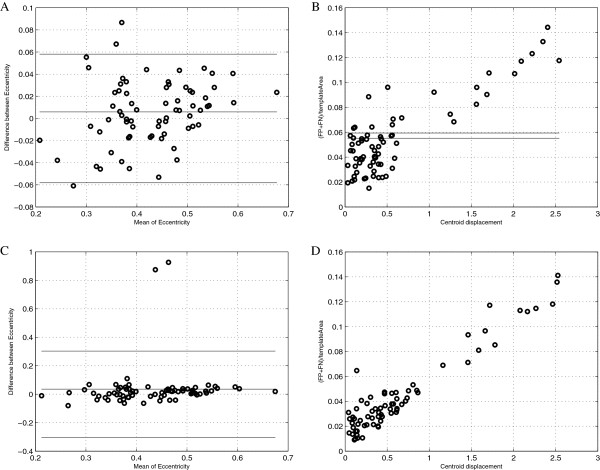
**The co-localization features.** The co-localization features: eccentricity (**A, C**) and defined by authors quasi B-A plots (**B, D**). **(A)** Bland-Altman plot of eccentricity, Bernsen method of segmentation on image after colour deconvolution; **(B)** quasi B-A plot (described in section “The methods of comparison of the chosen segmentation methods results”) Bernsen method of segmentation on image after colour deconvolution; **(C)** Bland-Altman plot of eccentricity, Sauvola method of segmentation on image after colour deconvolution; **(D)** quasi B-A plot, Sauvola method of segmentation on image after colour deconvolution.

The choice among previously mentioned methods and/or among the shape determined object filtration are examined based on B-A plots comparing shape features: perimeter, solidity, roundness and axis ratio, and two features which describe relative position (co-localization) of segmented and template objects: eccentricity and quasi B-A plots described further in this section. These quasi B-A plots show distribution of erroneously detected area (FP) as the function of the distance between centroids of selected and template objects. They have been calculated for all methods (6), all types of monochromatic image with various colour information (3) and all features (6), but only some of them, these which have impact in conclusions, are shown in Figure 6 and Figure 7.

B-A plots in Figure 6 present shape features (except axis ratio which results are similar to presented features): - solidity which shows if increase of objects’ size to achieve convex area is homogeneously distributed (Figure 6A,D,G), - roundness which shows if ratio of area to squared perimeter is independent from objects’ roundness (Figure 6B,E,F) and - perimeter length which shows if the changes in perimeter length compared to the template objects perimeter are independent from perimeter length (Figure 6C,F,I). All these features are presented for the Bernsen method applied to the image after colour deconvolution (Figure 6A,B,C) in the context of the plots of sum of the Bernsen and the White methods applied to blue channel of RGB image (Figure 6D,E,F). The first method plots present much more homogeneous distribution than the second group of plots which are presented below (respectively Figure 6D,E,F). These three shape features plots proof that error in object area detection (decrease of object size described above) for the Bernsen method applied to image after colour deconvolution is homogeneously distributed around object and do not affect its shape. Plots of B-A presented in Figure 6 (G, H, I) present also all previously described shapes coefficient for the Sauvola method applied to the result of image deconvolution. The values of false positive objects appear to be drastically different than the values of these coefficients for true positive objects. Based on this knowledge it is possible to form criteria (classifier) of false positive objects rejection from the set of results. So the Bernsen and the Souvola methods applied to result of deconvolution and shape coefficients (mainly solidity or perimeter) are the best candidates to be used in new hybrid method construction but only if the Bernsen method results of true positive objects indicate part of the Souvola method results.

B-A plots in Figure 7 presents co-localization features: eccentricity (Figure 7A,C) and defined by authors new coefficient (Figure 7B,D) which shows if the distance between two centroids is correlated with the ratio of the sum of false negative and false positive pixels divided by true positive pixels. Eccentricity defined as the ratio of the distance between the foci of the ellipse and its major axis length is calculated for ellipse that has the same second-moments as an object. Homogeneous distribution of error without any bias both for the Sauvola and the Bernsen method for eccentricity is achieved. It shows that erroneously detected area in both cases does not cause significant changes in ellipse which is an estimate of object. As this information do not tell us if errors in detected area moves centroid position more than within circle of reduce equal 1 pixel the new B-A like plots have been analysed. These plots are presented in Figure 7 (B, D) and they show that fraction of object which in consequence of error in peripheral part detection moves centroid of segmented object in comparison to the corresponding template object of distance between 1 and 2.5 pixels is less than 20% of objects (for the Bernsen method 12 objects from 70 but for the Sauvola method 14 objects from 72). So in most results of the Bernsen and the Souvola methods the error in area detection is homogeneously located on peripheral part of object if we applied these method to the monochromatic image after colour deconvolution. It proofs that the Brensen method results can be used as true positive objects markers (particularly if they are eroded using mathematical morphology operation [49,50]) and these markers should indicate inside of some of the Sauvola method results; all objects which are not marked are FP objects and can be rejected.

**Figure 8:**
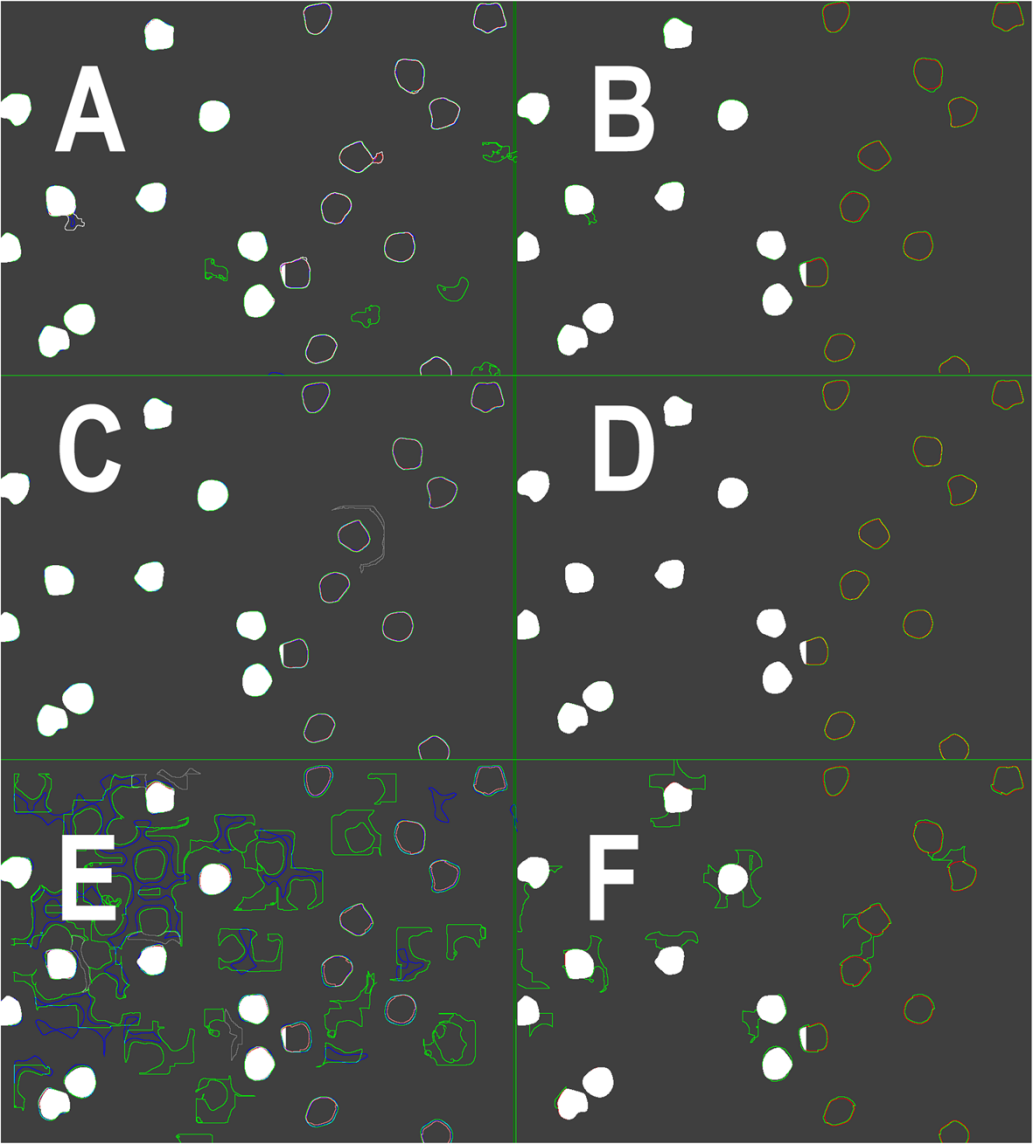
**Image segmentation results.** The sub-images present overlapped results of adaptive threshold methods in the left column for: the Niblack method (in red), the Sauvola method (in blue), the Yasuda method (in green) and the Palumbo method (in gray) and in the right column for: the White method (in red) and the Bernsen method (in green). The top row (**A** and **B**) presents results calculated on B-channel of RGB, the middle row (**C** and **D**) presents results calculated for the brown map after colour deconvolution while the bottom row (**E** and **F**) for the “brown axis” in RGB. The other colours appearing in image should be identified according to the law of primary colour adding as overlapping outlines. The left part of each sub-image is imposed on the template what causes that inside of object there is white colour while the right part, shows the mutual localization of the detected lines on dark gray instead of black background.

Figure 8 presents segmentation results calculated for the chosen fragment of image shown in Figure 3 (top-left) more detail for all types of the monochromatic images: in the first raw for B-channel, in the second raw for the result of deconvolution and the bottom raw for the results of brown component extraction. These results are presented as the various colour outlines of the detected objects. In left column of Figure 8 there are results of four methods: (1) Niblack method, in red colour, (2) Yasuda method, in green colour, (3) Palumbo method, in gray colour, and (4) Sauvola method, in blue colour. While in the right column there are only two: (1) White method, in red colour, and (2) Bernsen method, in green colour. Other colours which appear in image arising by the low of primary colour adding only for the overlapping outlines: yellow colour as result of green colour added to red colour, magenta colour as result of blue colour added to red colour, cyan colour as result of green colour added to red colour and white colour as result of adding all tree colours. The left part of each image is imposed on the template, while the right part, without the template. Both parts show the mutual localization of the detected lines relative to each other and to the template objects. Visual evaluation of the Figure 8 shows that template cover almost all detected objects outlines because detected object are smaller o just in size of template object so the difference of particular method results can be observed in right part of each image. All white pixels in left parts of all images and all yellow pixels in right parts shows agreement in selected outlines while the lines in other colours shows distance between results. These distances are relatively small for results of the segmentation performed with monochromatic image which is results of deconvolution and which is B-channel image (Figure 8A-D). There is presented only one FP object segmented by the Sauvola method in Figure 8C while in Figure 8A there are much more FP objects (in green colour) segmented by the Yasuda method. So all method of results comparison strengths our belief that the process of colour deconvolution produce monochromatic image with best performance of brown colour component.

## Discussion and conclusions

The investigation presented in this paper has two aims: (1) to compare the chosen adaptive threshold method on immunohistochemically stained lymphoma tissue sections to collect the knowledge how to design the new method based on the local thresholding methodology, and (2) to prove usefulness of creating artificial images which simulate experimentally acquired microscopic images used for the objective validation of image processing methods. The first goal has been achieved because results of all tested adaptive threshold methods except for the Yasuda method appear to be good or very good (accuracy from 0.9986 to 0. 9816 and precision from 1 to 0.6773 for respectively the Bernsen method and the Palumbo method applied to B-channel and to the White method applied to B-channel and for the Palumbo method applied to the result of the colour deconvolution) when accuracy and precision are quantifying based on pixels classification. The best accuracy and precision (respectively 0.9945 and 1) is for the White method applied to B-channel of RGB but this method decreases the size of segmented objects and sometimes reject objects that touches image edges. The accuracy and precision for both chosen methods are 0.9892 and 0.9331 for the Sauvola method and 0.9864 and 0.8454 for the Bernsen method calculating it from an area. But calculating it based on the number of selected objects for the Bernsen method accuracy and precision both are equal to 1 while for the modified Sauvola method are equal 1 and 0.9722 respectively.

All tested methods produce results based on various criteria but all uses the same size of the sliding window of image processing algorithm around classified pixels (in this investigation window size is 51*x*51 pixels because of object size) and the same value of minimal contrast for object and background (in this investigation *T*_*c*_=150): 

•the Bernsen method uses only these two parameters but it generally produces various threshold level across image plane, adjusting it to the mean value of two numbers: the maximum and the minimum of intensity in window; if local contrast is bigger than Tc the threshold value is settled on the level on locally adjusted value if not the background is detected;

•the hybrid of Sauvola method classifies objects according to description above using two other parameters: *k*=−0.2 which introduce bias in variance value and *R*=128 which allows to standardize variance value; this method also produce locally adjusted threshold level according to mean intensity value in window corrected by biased and standardized variance; if local contrast is bigger than Tc the threshold value is settled on the level on locally adjusted value if not the background is detected;

•the White method classifies object also according to pixels mean intensity value inside window but classifies it as belonging to the object if intensity of analyzed pixel multiplied by bias parameter (in this investigation *bias*=2) is bigger than mean intensity value calculated inside window, what is essential in this method that threshold level is also locally adjusted but local threshold value is dependent from mean intensity value in window and from chosen constant bias.

The local threshold level in the White method is dependent on bias which increase intensity of analysed pixel causes that the method perform well in images with high contrast between objects and background. The highest contrast is observed in blue channel monochromatic image despite the fact that texture present in blue objects locally disturbs this contrast. The other two methods are dependent on mean intensity corrected by the variance for the Sauvola method and on the half of intensity range inside window for the Bernsen method what causes that they are less dependent from the value of contrast but rather dependent from lack of local contrast disturbance. This is observed in monochromatic images after deconvolution where texture of blue object is rejected and texture in background is really weak. Both methods applying to the images after colour deconvolution produce complementary results. It derives from the fact that the corrected by standardized variance mean value of the intensity is sensitive enough to detect less conduced brown colour regions. It means that it can detect blurred edges of objects and at the same time it detects gentle contrails of stain deposits in the background while the half of the intensity range cut all blurred fragments of objects and do not detect stain deposits in the background. So it leads to the conclusion that the new developed method should take advantage from both the Bernsen and the Souvola methods in precision and accuracy of object detection and working synergistically it rejects all errors e.g. extra objects.

The evaluation of performance of 6 adaptive threshold methods, on three types of monochromatic images, based on 5 true colour artificial images was done. So the second aim, the verification of the thesis about usefulness of the artificial image synthesis method in the image processing method evaluation and comparison, also was achieved. The known and assumed location of objects of interest in the template allows using the standard methods for the quality assessment, as specificity, sensitivity and standard coefficients of similarity, precision and accuracy and Bland-Altman analysis which work well in all comparative study. As the scientific and clinical interest in quantifying brown objects in DAB&H stained samples is evident the evaluation of the segmentation results using artificial synthesized images allows gathering huge amount of knowledge about image analysis efficiency in the context of image characteristics. This knowledge will be used during new method development in future.

## Competing interests

The authors declare that they have no competing interests.

## Authors’ contributions

AK suggested the idea of investigation (artificial image and adjusted adaptive threshold method), wrote part of the paper with results, discussion and conclusions and some figures. LR wrote part of paper and tables and figures and designed and implemented software for image analysis and processing and Blend-Altman analysis, performed image analysis and consulted the obtained results. LW supported software implementation. CL, RB, ML performed experiments and acquired images and consulted the obtained results. All authors have read and approved the final manuscript.
